# A Comprehensive Review of Integrated Hall Effects in Macro-, Micro-, Nanoscales, and Quantum Devices

**DOI:** 10.3390/s20154163

**Published:** 2020-07-27

**Authors:** Avi Karsenty

**Affiliations:** 1 Advanced Laboratory of Electro-Optics (ALEO), Department of Applied Physics/Electro-Optics Engineering, Lev Academic Center, 9116001 Jerusalem, Israel; karsenty@jct.ac.il; Tel.: +972-2-675-1140; 2 Nanotechnology Center for Education and Research, Lev Academic Center, 9116001 Jerusalem, Israel

**Keywords:** Hall effect, nanoscale, microscale, macroscale, quantum-based devices, sensors, amplifiers, modeling, simulations, review

## Abstract

A comprehensive review of the main existing devices, based on the classic and new related Hall Effects is hereby presented. The review is divided into sub-categories presenting existing macro-, micro-, nanoscales, and quantum-based components and circuitry applications. Since Hall Effect-based devices use current and magnetic field as an input and voltage as output. researchers and engineers looked for decades to take advantage and integrate these devices into tiny circuitry, aiming to enable new functions such as high-speed switches, in particular at the nanoscale technology. This review paper presents not only an historical overview of past endeavors, but also the remaining challenges to overcome. As part of these trials, one can mention complex design, fabrication, and characterization of smart nanoscale devices such as sensors and amplifiers, towards the next generations of circuitry and modules in nanotechnology. When compared to previous domain-limited text books, specialized technical manuals and focused scientific reviews, all published several decades ago, this up-to-date review paper presents important advantages and novelties: Large coverage of all domains and applications, clear orientation to the nanoscale dimensions, extended bibliography of almost one hundred fifty recent references, review of selected analytical models, summary tables and phenomena schematics. Moreover, the review includes a lateral examination of the integrated Hall Effect per sub-classification of subjects. Among others, the following sub-reviews are presented: Main existing macro/micro/nanoscale devices, materials and elements used for the fabrication, analytical models, numerical complementary models and tools used for simulations, and technological challenges to overcome in order to implement the effect in nanotechnology. Such an up-to-date review may serve the scientific community as a basis for novel research oriented to new nanoscale devices, modules, and Process Development Kit (PDK) markets.

## 1. Introduction

### 1.1. Hall Effects—Brief History and Evolution

The Hall Effect is a well-known and established phenomenon since it was discovered by Edwin Herbert Hall (1855–1938) in 1879 [1,2], while he was a graduate student under the supervision of Henry Rowland (1848–1901) at the Physics Department of Johns Hopkins University. One hundred years later, on 13 November 1979, a symposium was held at Johns Hopkins University to commemorate the l00th anniversary of the discovery, and to present an up-to-date status of latest developments [3]. Consequently, a review proceeding was published to summarize the existing applications and components of the time [4]. While this was the status for the macro scale in the eighties, good progress occurred in the next decades, when additional related Hall Effects were discovered, enabling new opportunities. In fact, one can observe that there is not one unique Hall Effect, as per basic knowledge, but a series of Hall related phenomena, all distinguished by the dimensions and the context in which they are analyzed.

**Figure 1:**
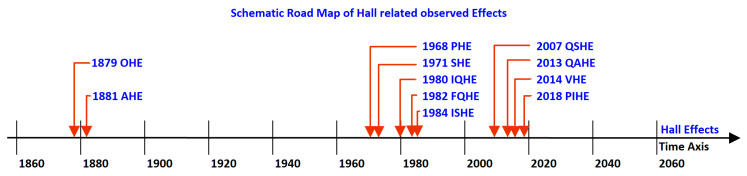
Schematic time line of the main Hall related observed Effects. OHE—Original Hall Effect; AHE—Anomalous Hall Effect; SHE—Spin Hall Effect; IQHE—Integer Quantum Hall Effect; FQHE—Fractional Quantum Hall Effect; ISHE—Inverse Spin Hall Effect; QSHE—Quantum Spin Hall Effect; QAHE—Quantum Anomalous Hall Effect; PHE—Planar Hall Effect; VHE—Valley Hall Effect; PIHE—Photo-Induced Hall Effect.

The Hall Effect, also called Original or Ordinary Hall Effect (OHE), can be used to distinguish currents that are composed of positively charged particles from those that are composed of negatively charged particles, as emphasized by Lorentz [5]. In 1880 and 1881, the Anomalous Hall Effect (AHE) [6] was observed on ferromagnetic materials by Hall himself [7,8]. If these effects were primarily discovered and applied at the macroscale level, other Hall related effects appeared at the microscale level over the years. Indeed, one hundred years later, in 1971, the Spin Hall Effect, was reported and the integer Quantum Hall Effect (QHE) [9,10], in 1980. More recently observed and understood, the Quantum Spin Hall Effect (QSHE) [11,12], in 2007, and the Quantum Anomalous Hall Effect (QAHE) [13,14,15,16], in 2013. Previous references, focused only on the observed physics phenomena, but not on the possible applications, and in particular on the potential devices that can be designed using such phenomena. Figure 1 presents a schematic timeline of the top ten discovered Hall related effects. If almost one century separates between the discovery of the original Hall Effects (OHE and AHE) and the quantum-based ones (mainly SHE and QHE) a shorter time frame of thirty years separates between the discovery of more complex phenomena (QSHE and QAHE).

### 1.2. Hall Effect Integration in Design Levels: Devices, Circuitry, and Process Development Kits

Moving away from Theoretical Physics to Applied Physics, or from phenomena study to concrete device applications, it appears that with time, the idea of using and moving the Hall Effect from macro to micro and nanoscales caught the attention of various research initiatives. Several types of devices sharing the Hall Effect were studied thoroughly in the past six decades. This is why, in addition to classic text books [17] mostly published several decades ago, and mainly focusing on OHE Physics, a series of new books appeared at the beginning of the twenty-first century, focusing this time on QHE Physics [18,19,20,21,22,23,24,25,26] and QSHE [27]. With the challenging attempts to integrate the Hall Effect phenomenon into devices, circuitry, and Process Development Kits (PDK) in mind, several well-structured books [28,29,30], industrial manuals [31], and review papers [32] were published along the years, including the theoretical description of tens of interesting and feasible applications. In the 1990s, partial reviews were performed in specific domains, such as the study of Hall Effect and Magnetoresistance Measurements in GaAs Materials and Devices [33]. Again, most of these important analyses were published a few decades ago, and were specialized and limited to specific domains. Moreover, some of the presented devices and systems are now obsolete since largely replaced by digital electronics solutions. It is in this context that an up-to-date review, extended to all domains, and oriented to the nanotechnology era, is more than called for.

**Figure 2:**
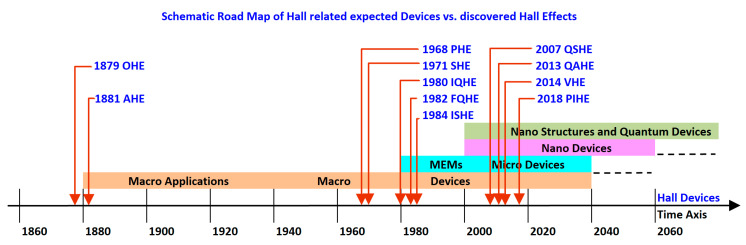
Enhanced schematic time line of the main Hall related observed Effects vs. the integrated devices.

Looking into previous publications, one can understand how useful the Hall Effect can be whenever applied into specific applications. In these text books, there are extensive lists of specialized devices. Let us look at two examples: In the sixties, W. E. Bulman presented a set of classic applications and devices, in a large study [34], and per different configurations. He divided the areas of applications to three parts: Magnetic fields measurement, magnetic fields control, and microwave power sensing. In case of combined Hall Effect probes and additional electromagnets, a series of macro devices could be obtained. Among others, one can find isolators, gyrators, transducers, circulators, phase detectors, but also magnetometers and magnetic tape read-out heads. A few decades later, in the 1990s, and still fitting the macroscale dimensions, Honeywell Corporate presented specialized devices applied to the sensing of parameters and applications [31]. Among others, and as a key player in the sensing activities, they presented Hall Effect-based sensors for the measurement of physical parameters such as flow rate, current, temperature, pressure, speed, angle, rounds per minute (RPM), position, etc. They proposed devices for diverse applications related to the civil life such as office machine sensor, magnetic card reader sensors, door interlock, and ignition sensors. Other devices were more oriented to the industry itself, such as automotive sensor, brushless DC motor sensor, piston detection sensor, transmission mounted speed sensors, etc. Dealing for years with the development of nanoscale devices, these two examples reinforced me in understanding how today, in the nanotechnology era, it became desirable to design nanoscale devices with an integrated Hall Effect, as presented all along this up-to-date review. Figure 2 presents a schematic flow of parallel progress, with the development of devices in macro-, micro- and nanoscale ranges depicted on a time axis of Hall Effects discoveries.

### 1.3. Hall Effect-Based Devices—Why?

There are several advantages of Hall Effect-based devices. High functionality and performance, adequate/good quality and reliability, large range of temperatures (−40 to +150 °C), and low cost are only part of these benefits. Moreover, Hall Effect provides galvanic isolation, meaning, and contactless sensing, which are crucial in many ways. Several decades ago, a long list of general features and desirable specifications was published for the macroscale Hall Effect-based sensing devices [31]. Today micro- and nanoscale enable more advanced applications. Even a simple everyday act of turning-off a smartphone screen by closing its wallet case is based on a Hall Effect sensor. Due to the technological progress over time, the reason to use a Hall Effect-based device also changed. For example, an updated and accurate list of advantages and disadvantages, first published in 2012, was updated and presented in 2019 [35]. Hall Effect Sensors (HES), share additional relevant advantages. From the quality and reliability point of view, the wear-out of such devices is usually low with time. In addition, external conditions such as vibrations, humidity or dust do not affect long-term functionality. Among the disadvantages is the limitation of the working distance between the sensor and the magnet. Moreover, since HES use the principle of a magnetic field, one can expect external magnetic fields to interfere and bias a current flow measurement. The temperature may affect the element’s electrical resistance of the carriers’ mobility and, as a consequence, its sensitivity. In spite of these weaknesses, Hall related effects present a huge interest to the industry.

## 2. Hall Effects Family Principles—Brief Qualitative Review

### 2.1. OHE—Original Hall Effect, 1879

**Figure 3:**
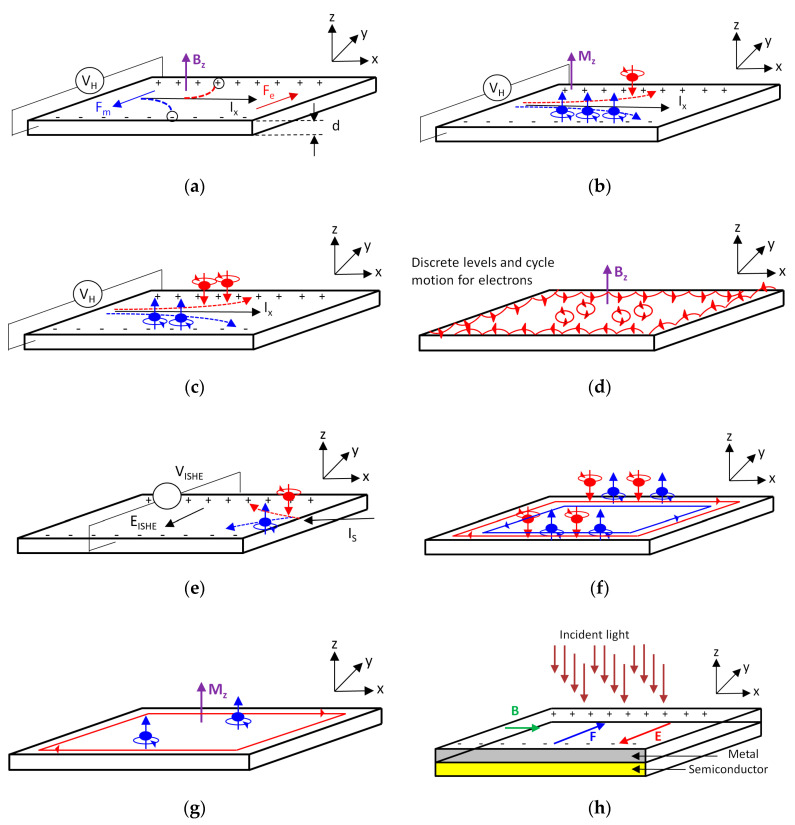
The main known Hall Effects and the year of their publication. (**a**) Original Hall Effect (OHE) [1], 1879; (**b**) Anomalous Hall Effect (AHE) [6], 1881; (**c**) Spin Hall Effect (SHE) [41,42], 1971; (**d**) Quantum Hall Effect (QHE) [9,10], 1980; (**e**) Inverse Spin Hall Effect (ISHE), 1984 [50,62,63]; (**f**) Quantum Spin Hall Effect (QSHE) [11,12], 2007; (**g**) Quantum Anomalous Hall Effect (QAHE) [13,14,15,16], 2013; (**h**) Photo-Induced Hall Effect (PIHE), 2018 [58].

The Original Hall Effect is presented in Figure 3a. At equilibrium, a voltage difference called the Hall Voltage (HV) appears when, a magnetic field is applied upon an electrical conductor cross by an electric current. The HV becomes maximal when the magnetic field is perpendicular to the current. According to Figure 3a, as the free carriers travel along the current direction Ix through the conductor, lying itself in a perpendicular magnetic field Bz, they will be influenced by a magnetic force, which will drive them to move in the Y direction and accumulate on one side of the conductor. This charge separation leads to an electric field E_Y_ and consequently to a potential difference. The charge builds up until the electric force induced by the electric equilibrates the magnetic force. Then, the steady Hall Voltage can be measured and is found proportional to both the electric current and the magnetic field as shown in Section 3.

DC magnetic fields are traditionally used to extract the mobility of the majority of free carrier from the Hall Voltage, as described in Section 3 below. However, the measurement error may appear due to a misalignment between the contacts, leading to parasitic voltage, found proportional to the current, and the material resistivity. Though this kind of offset voltage can be reduced by reversing the current and the magnetic field, it is more effective to use an AC magnetic field, especially for low mobility material (<1 cm^2^/Vs). In this case, the mobility can be extracted accurately by means of a lock-in technique [36,37,38].

### 2.2. AHE—Anomalous Hall Effect, 1881

In 1881, two years after he discovered the OHE, Hall reported that the effect was ten times larger in ferromagnetic conductors than in non-magnetic conductors. This new effect, entitled “Anomalous” Hall Effect (AHE), is presented in Figure 3b. As per Nagaosa et al. [6], both discoveries were remarkable, given the limited knowledge at that time on how free carriers’ move through conductors. The first discovery, OHE, provided an elegant tool to measure carriers’ concentration more accurately in non-magnetic conductors, and introduced the semiconductor physics and solid-state electronics in the late 1940s. For a long period, AHE remained an enigmatic problem to explain since it involves topology and geometry concepts, which have been formulated only in the last decades. Only after the Berry phase approach was adopted [39], it was possible to link between the topological nature of the Hall currents and the AHE itself. In nanoscale systems, where a priori direct measurements are not straight forward and sometimes not possible, the AHE can serve as a useful probe of electron spin polarization [40].

### 2.3. SHE—Spin Hall Effect, 1971

The Spin Hall Effect (SHE), presented in Figure 3c, is a spin transport phenomenon theoretically predicted by Dyakonov and Perel in 1971 [41,42]. It causes the scattering and accumulation of spins of opposite signs at opposing lateral edges of a sample induced by a longitudinal charge current. Since the SHE is a purely spin-based phenomenon the current carried by the sample will generate a transverse polarized spin-current though no net charge current. Since this effect does not require time-reversal (TR) symmetry breaking, it can occur without any magnetic field. SHE was confirmed experimentally for the first time in 2004 on GaAs and InGaAs semiconductors at 30 K [43].

### 2.4. IQHE—Integer Quantum Hall Effect, 1980

In regard to the Quantum Hall Effect, presented in Figure 3d, it would be wiser to refer to the two parts separately: The Integer Quantum Hall Effect (IQHE) and the Fractional Quantum Hall Effect (FQHE). Discovered approximately 100 years after Hall’s initial work, and first published in 1980, by Von Klitzing [44], the IQHE effect was observed in a 2D electron system, located at Si/SiO_2_ interface or a Field Effect Transistor (MOS-FET). The device, sharing a Hall bar geometry, is placed into a strong magnetic field of about 15 T and at liquid helium temperature. This important research discovery granted Von Klitzing the 1985 Nobel Prize in Physics and led to the adoption of internationally accepted rules for resistance calibration [45,46]. The IQHE is obtained by varying the gate voltage in such a way that the Hall resistance varies stepwise by values of *h/ie^2^* (*i* is an integer) while *h* is the Planck constant and *e* the electron charge. The *i* step indicates the filling of a Landau level corresponding to a quantized cyclotron orbit of the electron in a magnetic field. As explained explicitly by Prof. D. Tong in his recent lectures at Cambridge University [47], the origin of these plateaux is related to impurities, creating “disorder” and causing a split that degenerates eigenstates of the electron wave functions. In fact, such quantum phenomenon can be explained without considering the interactions between electrons, assuming that there are quantum states for a single particle in a magnetic field. More recently, in 2007, the integer quantum Hall Effect was reported in graphene at room temperature [48].

### 2.5. FQHE—Fractional Quantum Hall Effect, 1982

The Fractional Quantum Hall Effect (FQHE) was observed for the first time, and reported in 1982, by Tsui et al. [49] in a 2D high electron mobility GaAlAs heterostructure at liquid helium temperature. When compared to the Integer Quantum Hall Effect, the Fractional Quantum Hall Effect (FQHE) presents, additional plateaux of Hall resistance at fractional values of *i* = 1/3, 2/3, and 3/2 in the *h/ie^2^* expression. A decade later, in 1998, Tsui was the recipient of the Physics Nobel Prize along with Laughlin and Stromer, for “Their discovery of a new form of quantum fluid with fractionally charged excitations”. Indeed, in the FQHE, electrons are expected to bind together with magnetic flux lines and make new quasiparticles, also called “composite fermions”, paving the way to a new quantum state of matter as described in more detail by D. Tong in his lectures [47].

### 2.6. ISHE—Inverse Spin Hall Effect, 1984

The Inverse Spin Hall Effect (ISHE), presented in Figure 3e, was first evidenced by Bakun et al. [50] in 1984 through experimental observations of a spin-orbit induced photocurrent on AlGaAs crystals at 77 K. If for the SHE, only a spin current is detected, in the ISHE reciprocal effect, coupling of the spin-current can generate a transverse charge current. In 2014, through their deep analysis, Sinova et al. [51] largely explained the difference between the SHE and ISHE. However, as exactly defined by Boehme in 2016 [52,53], “the inverse spin Hall Effect is a remarkable phenomenon that turns so-called spin current into an electric current. The effect is so odd that nobody really knows what this will be used for eventually, but many technical applications are conceivable, including very odd new power-conversion schemes”.

### 2.7. QSHE—Quantum Spin Hall Effect, 2007

The Quantum Spin Hall Effect, presented in Figure 3f, was observed experimentally in 2007 in CdTe/HgTe Quantum Wells [54,55]. This effect is linked to the QHE predecessor, but is also quite different. In fact, new topological states, called Quantum Spin Hall (QHS) states are characterized by a quantized spin-Hall conductance and a vanishing charge-Hall conductance. Unlike Landau levels of the QHE, the existence of QSH states does not require a large magnetic field. Indeed, since these states are Time Reversal (TR) invariant, there is no need for an external field to break the TR symmetry.

### 2.8. QAHE—Quantum Anomalous Hall Effect, 2013

In the case of the Quantum Anomalous Hall Effect (QAHE), presented in Figure 3g, the phenomenon is running in a system without any external applied magnetic field, as described clearly only recently by Liu et al. [56]. Defined as a quantized Hall Effect, it represents a new appearance of topological structure in many-electrons systems, and sharing a high potential of possible applications in electronic devices. More recently, the effect was largely investigated, theoretically modeled, and physically experimented [57].

### 2.9. PIHE—Photo-Induced Hall Effect, 2018

At the end, a new effect, entitled Photo-Induced Hall Effect (PIHE) and presented in Figure 3h, was recently proposed and reported by Li and Rutuolo [58]. In this new technique and setup configuration, one creates a photo-induced effect in metals, dedicated to bias-free magnetic sensing. The idea is to overcome the existing limitation of the Original Hall Effect (OHE) in metals, since the effect found there is sometimes too small to enable practical applications when compared to the same effect in semiconductors, where it is a standard for magnetic field sensing. In this setup, there is a transparent metal forming a Schottky contact to a semiconductor. The obtained injection of charge is the result of an incident light trigger, reaching the interface, from a space charge region. Whenever a magnetic field is applied, then a voltage, which is proportional to the field, as well as the light intensity, appears at the metal edges. By illuminating the metal, photo-induced charges are produced and injected from the space charge region. By applying a magnetic field a transverse open circuit voltage appears at the contact edges and is proportional to the light intensity and to the magnetic field. As described in Figure 3h, the charges are deflected by a magnetic field in order to produce an electric one, which is perpendicular to both light and field directions.

With time, and in addition to the above mentioned nine main effects, several discovered concepts and experimental configurations were eventually named and received acronyms. Following is the description of a few. At the end, general electro-magnetism parameters and units have been summarized in Appendix A, while Hall Effect related acronyms have been summarized in Appendix B, and the complete review in Appendix C.

### 2.10. PHE—Planar Hall Effect, 1968

Among all the devices presented in this review, one is the planar Hall sensor, based on the Planar Hall Effect of ferromagnetic materials having an anisotropic magneto-resistivity. By measuring the magneto-resistance change, one can map the magnetic field components inside the sensor plane. The effect was already investigated in the 1960s by Vu Dinh Ky on Ni, Fe, Co, and Ni-Fe films with thicknesses between 10 and 150 nm in a range of temperatures varying from 77 to 293 K [59]. The rationale for naming this concept “Planar Hall Effect (PHE)”, lies in the fact that its basic characteristic behavior is opposed to the regular Hall sensor, which measures field components perpendicular to the sensor plane. For ferromagnetic materials, the Hall resistance depends on the orientation of the current relative to the magnetization direction. Consequently, such a property causes an asymmetric electric field perpendicular to the current, depending on the sensor magnetization orientation. When an external magnetic field is applied in the sensor plane it will change the direction of the magnetization. In such a way, the voltage read out of sensor will be changed linearly with the in plane magnetic field.

### 2.11. VHE—Valley Hall Effect, 2014

Mak et al. [60] observed the Valley Hall Effect (VHE) in a monolayer of MoS_2_. In a two-dimensional material, the electronic structure enables distinguishing two separated valleys of energy. The different valleys’ electrons move in opposite directions across the sample. Using different methods, there were several attempts to create inequity in the population of the two valleys. It seems that this domain can become an emerging field of “valleytronics”, as expanded later by additional teams [61].

## 3. Review of Analytical and Numerical Models

While Section 3.1 and Section 3.2 deal with DC analytical models, Section 3.3, Section 3.4, and Section 3.5 present several AC models. In the DC models, Section 3.1 presents an isotropic approach (i.e., Hall Effect in one direction), and Section 3.2 an anisotropic approach (i.e., few directions). The five sections are all classic models. The quantal Hall Effect models are still in the process of investigation and are not fully exploitable yet in numerical tools. Section 3.6 completes the picture with a survey of the main numerical complementary Best Known Methods (BKM).

### 3.1. Classical Hall Effect

When compared to new models, dealing with additional nanoscale concerns and considerations, the classical approach of the Hall Effect is based on a well-known set of assumptions and equations. However, in order to present and analyze several advanced case studies later on in this review, it is necessary to return to the basics. Assuming a free carrier at equilibrium, and also assuming the following definitions:

L_y_ is the transverse width of the Hall Bar; A_x_ = L_y_.d is the transverse cross sectional area of the Hall Bar; v_D_ is the drift velocity of the free carrier; B_z_ is the magnetic field in the Z direction; E_y_ is the electric field in the Y direction; V_H_ is the Hall Voltage; V_Y_ is the voltage in the Y direction; I_x_ is the electric current in the Hall Bar; q is the elementary charge; and n is the free electron density. We get:

(1) $${\vec{F}}_{\mathrm{electric}}={\vec{F}}_{\mathrm{magnetic}}$$

After axis projection (Figure 3a), we get:

(2) $${q\vec{E}}_{Y}={q\vec{v}}_{D}\times {\vec{B}}_{Z}$$

And also:

(3) $$\left\|{\vec{E}}_{Y}\|=\|{\vec{v}}_{D}\|\|{\vec{B}}_{Z}\right\|$$

Since:

(4) $$V_{Y}=E_{Y}\cdot L_{y}$$

The Hall Voltage V_Y_ is defined as V_Y_ potential at equilibrium. Therefore, from Equations (3) and (4) we obtain Equation (5):

(5) $$V_{Y}=E_{Y}\cdot L_{y}$$

And since:

(6) $$v_{D}=\frac{I_{x}}{A_{x}\mathrm{qn}}$$

We get:

(7) $$V_{H}=\frac{I_{x}B_{Z}L_{y}}{A_{x}\mathrm{qn}}$$

The Hall magneto resistance is defined as the ratio of the electrical voltage in the Y direction over the current in the X direction:

(8) $$R_{\mathrm{xy}}=\frac{V_{Y}}{I_{x}}=R_{H}B_{Z}\frac{L_{y}}{A_{x}}\;,$$

R_H_ is the 3D Hall coefficient, and is defined as:

(9) $$R_{H}=\frac{1}{\mathrm{nq}},$$

### 3.2. DC Hall Magneto-Resistance

For this new model, it is necessary to use the Drude model [64]. This approach enables a good assessment of the resistance. Moreover, such model is applicable in case scattering understanding is independent of the nature of the carrier scattering mechanism. This brings us to the motion’s equation for the momentum per free carrier:

(10) $$\frac{d\vec{p}}{\mathrm{dt}}=-\frac{\vec{p}}{\tau }+\vec{F}$$

where p is the momentum per free carrier, τ is the collision time (mean free time), and F is the external force. Generally, the velocity of the free carrier is:

(11) $$\vec{v}=v_{x}\hat{x}+v_{y}\hat{y}+v_{z}\hat{z}$$

Assuming the electric field is:

(12) $$\vec{E}=E_{x}\hat{x}+E_{y}\hat{y}$$

and the magnetic field is:

(13) $$\vec{B}=B_{z}\hat{z}$$

So the equation of motion is:

(14) $$m_{e}\left(\frac{d}{\mathrm{dt}}+\frac{1}{\tau }\right)\vec{v}=q\left(\vec{E}+\vec{v}\times \vec{B}\right)$$

where m_e_ is the effective mass of the free carrier, and q is the charge of the carrier and equals to +e for hole and −e for electron. In case of DC electrical conductivity, we obtain the two following equations:

(15) $$F_{x}=m_{e}\left(\frac{d}{\mathrm{dt}}+\frac{1}{\tau }\right)v_{x}=q\left(E_{x}+v_{y}B_{z}\right)$$

(16) $$F_{y}=m_{e}\left(\frac{d}{\mathrm{dt}}+\frac{1}{\tau }\right)v_{y}={\mathrm{qv}}_{x}B_{z}$$

In steady state condition:

(17) $$\frac{m_{e}v_{x}}{\tau }=q\left(E_{x}+v_{y}B_{z}\right)\to v_{x}=\frac{q\tau E_{x}}{m_{e}}+\omega_{c}v_{y}\tau$$

(18) $$\frac{m_{e}v_{y}}{\tau }=q\left(E_{y}-v_{x}B_{z}\right)\to v_{y}=\frac{q\tau E_{y}}{m_{e}}-\omega_{c}v_{x}\tau$$

where:

(19) $$\omega_{c}=\frac{{\mathrm{qB}}_{z}}{m_{e}}\;\mathrm{is}\;\mathrm{the}\;\mathrm{cyclotron}\;\mathrm{frequency}\;(\mathrm{in}\;\mathrm{Hz}\;\mathrm{units})$$

In steady state, since:

(20) $$v_{y}=0$$

Then:

(21) $$E_{x}=-m_{e}\frac{v_{x}}{q\tau }$$

And:

(22) $$E_{y}=m_{e}\frac{\omega_{c}v_{x}}{q}$$

Therefore:

(23) $$E_{y}=-\omega_{c}{\tau E}_{x}$$

In the presence of magnetic field, the resistivity tensor is defined according to:

(24) $$\left(\begin{array}{c} E_{x}\\ E_{y} \end{array}\right)=\left[\begin{array}{cc} \rho_{\mathrm{xx}} & \rho_{\mathrm{xy}}\\ \rho_{\mathrm{yx}} & \rho_{\mathrm{yy}} \end{array}\right]\left(\begin{array}{c} j_{x}\\ j_{y} \end{array}\right)$$

Indeed, according to Equation (17):

(25) $$j_{x}={\mathrm{qnv}}_{x}=\frac{{\mathrm{nq}}^{2}\tau }{m_{e}}E_{x}+{\mathrm{qn}\omega }_{c}v_{y}$$

By defining σ_0_ the intrinsic conductivity without magnetic field (for B_z_ = 0) as:

(26) $$\sigma_{0}=\frac{q^{2}n\tau }{m_{e}}$$

Then:

(27) $$j_{x}=\sigma_{0}E_{x}+\omega_{c}{\tau j}_{y}$$

In the same way, based on Equation (18):

(28) $$j_{y}={\mathrm{qnv}}_{y}=\sigma_{0}E_{y}+{\mathrm{qn}\omega }_{c}v_{y}$$

We get:

(29) $$\{\begin{array}{c} \sigma_{0}E_{x}=j_{x}-\omega_{c}\tau j_{y}\\ \sigma_{0}E_{y}=-\omega_{c}\tau j_{x}+j_{y} \end{array}$$

Then, the matrix equation appears as:

(30) $$\left(\begin{array}{c} E_{x}\\ E_{y} \end{array}\right)=\left[\begin{array}{cc} \frac{1}{\sigma_{0}} & \frac{\omega_{c}\tau }{\sigma_{0}}\\ -\frac{\omega_{c}\tau }{\sigma_{0}} & \frac{1}{\sigma_{0}} \end{array}\right]\left(\begin{array}{c} j_{x}\\ j_{y} \end{array}\right)$$

where the resistivity tensor coefficients are by identification to (27).

(31) $$\rho_{\mathrm{xx}}=\rho_{\mathrm{yy}}=\frac{1}{\sigma_{0}}=\frac{m_{e}}{q^{2}n\tau }$$

(32) $$\rho_{\mathrm{xy}}=-\rho_{\mathrm{yx}}=\frac{\omega_{c}\tau }{\sigma_{0}}=\frac{B_{z}}{\mathrm{nq}}=R_{H}B_{z}$$

Now we can define the conductivity tensor as:

(33) $$\left(\begin{array}{c} j_{x}\\ j_{y} \end{array}\right)=\left[\begin{array}{cc} \sigma_{\mathrm{xx}} & \sigma_{\mathrm{xy}}\\ \sigma_{\mathrm{yx}} & \sigma_{\mathrm{yy}} \end{array}\right]\left(\begin{array}{c} E_{x}\\ E_{y} \end{array}\right)$$

where:

(34) $$\left[\begin{array}{cc} \sigma_{\mathrm{xx}} & \sigma_{\mathrm{xy}}\\ \sigma_{\mathrm{yx}} & \sigma_{\mathrm{yy}} \end{array}\right]={\left[\begin{array}{cc} \rho_{\mathrm{xx}} & \rho_{\mathrm{xy}}\\ \rho_{\mathrm{yx}} & \rho_{\mathrm{yy}} \end{array}\right]}^{-1}$$

where:

(35) $$\sigma_{\mathrm{xx}}=\sigma_{\mathrm{yy}}=\frac{\rho_{\mathrm{xx}}}{\rho_{\mathrm{xx}}{}^{2}+\rho_{\mathrm{xy}}{}^{2}}=\frac{\sigma_{0}}{1+{\left(\omega_{c}\tau \right)}^{2}}=\frac{\sigma_{0}}{1+{\left(\sigma_{0}R_{H}B_{z}\right)}^{2}}$$

(36) $$\sigma_{\mathrm{xy}}=-\sigma_{\mathrm{yx}}=-\frac{\rho_{\mathrm{xy}}}{\rho_{\mathrm{xx}}{}^{2}+\rho_{\mathrm{xy}}{}^{2}}=-\frac{\sigma_{0}\omega_{c}\tau }{1+{\left(\omega_{c}\tau \right)}^{2}}=\frac{\sigma_{0}{}^{2}R_{H}B_{z}}{1+{\left(\sigma_{0}R_{H}B_{z}\right)}^{2}}$$

### 3.3. Dynamic Magneto-Conductivity Tensor for Free Carrier

In case of the oscillating magnetic field in *z* direction, the approximation for dynamic magneto-conductivity tensor for free carriers will require a new approach. For calculation, the model will combine both Perturbation Theory and the assumptions of the Drude model, as presented in the previous paragraph, for DC Hall magneto-resistance. As a disclaimer, it should be emphasized that ballistic conductance and Quantum Hall Effect will not be considered for the model, even though the latter becomes more relevant at strong magnetic fields (B > 0.5 T). Therefore, the equations of motion, for momentum per carrier will be as following:

(37) $$m_{e}\left(\frac{d\vec{v}}{\mathrm{dt}}+\frac{\vec{v}}{\tau }\right)=q\left(\vec{E}+\vec{v}\times \vec{B}\right)$$

where:

(38) $$\vec{v}={\vec{v}}_{0}+{\varepsilon \vec{v}}_{1}+\varepsilon^{2}{\vec{v}}_{2}$$

and

(39) $$\varepsilon =\frac{\left\|{\vec{v}}_{0}\|\|\vec{B}\right\|}{\left\|\vec{E}\right\|}\ll 1$$

is the perturbation term of the magnetic field.

V_1_ and V_2_ are respectively the first and the second perturbation terms of carrier velocity while V_0_ is the non-perturbed term (no magnetic field).

ε^1^ and ε^2^ are respectively the first and the second perturbation orders (1 and 2 are the exponents of the perturbation coefficient) of carrier velocity while ε^0^ = 1 is the non-perturbed coefficient. We used it to link the perturbation order to the velocity perturbed term.

**For zero order approximation (no magnetic perturbation) ε^0^ the equation becomes:**

(40) $$m_{e}\left(\frac{d{\vec{v}}_{0}}{\mathrm{dt}}+\frac{{\vec{v}}_{0}}{\tau }\right)=q\vec{E}$$

Since $\vec{E}$ is static, we get

(41) $$\frac{{d\vec{v}}_{0}}{\mathrm{dt}}=0$$

Therefore:

(42) $$m_{e}\frac{{\vec{v}}_{0}}{\tau }=q\vec{E}\to {\vec{v}}_{0}=\frac{q\tau }{m_{e}}\vec{E}\to {\vec{v}}_{0}=\mu_{e}\vec{E}$$

where µ_e_ is the free carrier effective mobility.

Since

(43) $$\vec{J}=qn\vec{v}$$

(44) $${\vec{J}}_{0}=\frac{{\mathrm{nq}}^{2}\tau }{m_{e}}\vec{E}=\sigma_{0}\vec{E}$$

**For first order ε^1^ the equation becomes:**

(45) $$m_{e}\left(\frac{{d\vec{v}}_{1}}{\mathrm{dt}}+\frac{{\vec{v}}_{1}}{\tau }\right)={q\vec{v}}_{0}\times \vec{B}$$

Since

(46) $$\vec{B}\left(t\right)={\vec{B}}_{0}e^{-i\omega t},\;{\vec{v}}_{1}={\vec{v}}_{10}e^{-i\omega t}$$

Therefore:

(47) $$m_{e}\left(-i\omega +\frac{1}{\tau }\right){\vec{v}}_{10}e^{-i\omega t}=-{e\vec{v}}_{0}\times {\vec{B}}_{0}e^{-i\omega t}$$

Since

(48) $${\vec{v}}_{0}=+/-\mu_{e}\vec{E}\;(+\mathrm{in}\;\mathrm{case}\;\mathrm{of}\;\mathrm{holes},\;-\;\mathrm{in}\;\mathrm{case}\;\mathrm{of}\;\mathrm{electrons})$$

(49) $$\left(1-i\omega \tau \right){\vec{v}}_{10}=\frac{q\tau \mu_{e}}{m_{e}}\vec{E}\times {\vec{B}}_{0}\to {\vec{v}}_{10}=\frac{\mu_{e}{}^{2}\vec{E}\times {\vec{B}}_{0}}{\left(1-i\omega \tau \right)}$$

Since

(50) $$\vec{J}=qn\vec{v}$$

(51) $${\vec{J}}_{10}=\frac{\sigma_{0}\mu_{e}{\vec{B}}_{0}\times \vec{E}}{\left(1-i\omega \tau \right)}=\left[\begin{array}{ccc} 0 & -\frac{\sigma_{0}\mu_{e}\|\vec{B}\|}{1-i\omega \tau } & 0\\ \frac{\sigma_{0}\mu_{e}\left\|\vec{B}\right\|}{1-i\omega \tau } & 0 & 0\\ 0 & 0 & 0 \end{array}\right]\vec{E}$$

(52) $${\vec{J}}_{10}=\left[\begin{array}{ccc} 0 & -\frac{\sigma_{0}\varepsilon }{1-i\omega \tau } & 0\\ \frac{\sigma_{0}\varepsilon }{1-i\omega \tau } & 0 & 0\\ 0 & 0 & 0 \end{array}\right]\vec{E}$$

**For second order ε^2^ the equation becomes:**

(53) $$m_{e}\left(\frac{{d\vec{v}}_{2}}{\mathrm{dt}}+\frac{{\vec{v}}_{2}}{\tau }\right)={q\vec{v}}_{1}\times \vec{B}$$

Since

(54) $${\vec{v}}_{1}\vec{B}={\vec{v}}_{10}{\vec{B}}_{0}e^{-i2\omega t}$$

and

(55) $${\vec{v}}_{2}={\vec{v}}_{20}e^{-i2\omega t}$$

Therefore:

(56) $$m_{e}\left(-i2\omega +\frac{1}{\tau }\right){\vec{v}}_{20}e^{-i2\omega t}=-{e\vec{v}}_{10}\times {\vec{B}}_{0}e^{-i2\omega t}$$

Since

(57) $${\vec{v}}_{10}=\frac{\mu_{e}{}^{2}\vec{E}\times {\vec{B}}_{0}}{\left(1-i\omega \tau \right)}$$

Then

(58) $$\left(1-i2\omega \tau \right){\vec{v}}_{20}=-\frac{q\tau }{m_{e}}\frac{\mu_{e}{}^{2}\left(\vec{E}\times {\vec{B}}_{0}\right)}{\left(1-i\omega \tau \right)}\times {\vec{B}}_{0}=-\frac{q\tau }{m_{e}}\frac{\mu_{e}{}^{2}{\vec{B}}_{0}}{\left(1-i\omega \tau \right)}\times \left({\vec{B}}_{0}\times \vec{E}\right)$$

Since

(59) $$\vec{J}=qn\vec{v}$$

Then

(60) $${\vec{J}}_{20}=\frac{\sigma_{0}\mu_{e}{}^{2}{\vec{B}}_{0}}{\left(1-i\omega \tau \right)\left(1-i2\omega \tau \right)}\times ({\vec{B}}_{0}\times \vec{E})$$

(61) $${\vec{J}}_{20}=\frac{\sigma_{0}\mu_{e}{}^{2}\|{\vec{B}}_{0}\|{}^{2}}{\left(1-i\omega \tau \right)\left(1-i2\omega \tau \right)}\left[\begin{array}{ccc} 0 & -1 & 0\\ 1 & 0 & 0\\ 0 & 0 & 0 \end{array}\right]\left[\begin{array}{ccc} 0 & -1 & 0\\ 1 & 0 & 0\\ 0 & 0 & 0 \end{array}\right]\vec{E}$$

(62) $${\vec{J}}_{20}=\frac{\sigma_{0}\mu_{e}{}^{2}\|{\vec{B}}_{0}\|{}^{2}}{\left(1-i\omega \tau \right)\left(1-i2\omega \tau \right)}\left[\begin{array}{ccc} -1 & 0 & 0\\ 0 & -1 & 0\\ 0 & 0 & 0 \end{array}\right]\vec{E}$$

(63) $${\vec{J}}_{20}=\frac{\sigma_{0}\varepsilon^{2}}{\left(1-i\omega \tau \right)\left(1-i2\omega \tau \right)}\left[\begin{array}{ccc} -1 & 0 & 0\\ 0 & -1 & 0\\ 0 & 0 & 0 \end{array}\right]\vec{E}$$

Since

(64) $$\vec{J}={\vec{J}}_{0}+{\vec{J}}_{1}+{\vec{J}}_{2}$$

and

(65) $$\vec{J}=\left[\sigma \right]\vec{E}$$

(66) $$\vec{J}=\left[\begin{array}{ccc} \sigma_{0}-\frac{\sigma_{0}\varepsilon^{2}}{\left(1-i\omega \tau \right)\left(1-i2\omega \tau \right)} & -\frac{\sigma_{0}\varepsilon }{1-i\omega \tau } & 0\\ \frac{\sigma_{0}\varepsilon }{1-i\omega \tau } & \sigma_{0}-\frac{\sigma_{0}\varepsilon^{2}}{\left(1-i\omega \tau \right)\left(1-i2\omega \tau \right)} & 0\\ 0 & 0 & \sigma_{0} \end{array}\right]\vec{E}$$

(67) $$\left[\sigma \right]=\left[\begin{array}{ccc} \sigma_{\mathrm{xx}} & \sigma_{\mathrm{xy}} & \sigma_{\mathrm{xz}}\\ \sigma_{\mathrm{yx}} & \sigma_{\mathrm{yy}} & \sigma_{\mathrm{yz}}\\ \sigma_{\mathrm{zx}} & \sigma_{\mathrm{zy}} & \sigma_{\mathrm{zz}} \end{array}\right]=\left[\begin{array}{ccc} \sigma_{0}-\frac{\sigma_{0}\varepsilon^{2}}{\left(1-i\omega \tau \right)\left(1-i2\omega \tau \right)} & -\frac{\sigma_{0}\varepsilon }{1-i\omega \tau } & 0\\ \frac{\sigma_{0}\varepsilon }{1-i\omega \tau } & \sigma_{0}-\frac{\sigma_{0}\varepsilon^{2}}{\left(1-i\omega \tau \right)\left(1-i2\omega \tau \right)} & 0\\ 0 & 0 & \sigma_{0} \end{array}\right]$$

### 3.4. Two-Dimensional Electron Gas (2DEG) and Heterodyne Hall Effect

When, combined to the Hall Effect, in such a way that both magnetic and electric fields are oscillating at resonant frequencies, we get an example of a heterodyne device. As reported recently by Oka and Bucciantini [65], a heterodyne device can be realized by applying an oscillating electric field acting as an input signal to a 2DEG and coupled with an oscillating magnetic field acting as a driving signal. Due to the Hall Effect, the current flowing perpendicularly to the applied electric field is found to be resonant at the input signal frequency shifted by integer multiples of the driving frequency. In such cases, we define j_a_(mΩ) the electric current density as the output signal, sharing a frequency mΩ, and flowing along the a-direction (a, b = x, y, z) and a weak electric field, in the b-direction, and frequency nΩ. In this case, the heterodyne conductivity is a four-index tensor [65], and the electric field is noted $E_{b}^{n}$.

(68) $$j_{a}\left(m\Omega \right)=\underset{b}{\sum }\sigma_{\mathrm{ab}}^{m,n}\left(n\Omega \right)E_{b}^{n}$$

It can be shown that for the classical case the heterodyne conductivity $\sigma_{\mathrm{ab}}^{m,n}$ for n = 0 (static electric field) [65] is:

(69) $$\sigma_{\mathrm{xy}}^{0,0}=0,\;\sigma_{\mathrm{yy}}^{0,0}=\sigma_{0}J_{0}{\left(r\right)}^{2}$$

(70) $$\sigma_{\mathrm{xy}}^{1,0}=\sigma_{0}\Omega \tau \frac{J_{0}\left(r\right)J_{1}\left(r\right)}{1+{\left(\Omega \tau \right)}^{2}},\;\sigma_{\mathrm{yy}}^{1,0}=\sigma_{0}\Omega \tau \frac{J_{0}\left(r\right)J_{1}\left(r\right)}{1+{\left(\Omega \tau \right)}^{2}},$$

With σ_0_ = q^2^n/(ηm) being the zero field expression of the conductivity. η is a small phenomenological scattering parameter. J_0_(r) is the zero order Bessel function; J_1_(r) is the first order of the Bessel function; Ω is the magnetic field frequency; ω_c_ is the cyclotron frequency; and r = ω_c_/Ω is the Bessel function argument.

### 3.5. Free Electron Model and Dielectric Tensor

The medium dielectric function tensor is related to the conductivity tensor as:

(71) $$\left[\epsilon \right]=1+i\left(\frac{1}{\in_{0}\omega }\right)\left[\sigma \right]$$

Therefore:

(72) $$\in =\left[\begin{array}{ccc} 1+\frac{i}{\in_{0}\omega }\sigma_{\mathrm{xx}} & \frac{i}{\in_{0}\omega }\sigma_{\mathrm{xy}} & 0\\ \frac{i}{\in_{0}\omega }\sigma_{\mathrm{yx}} & 1+\frac{i}{\in_{0}\omega }\sigma_{\mathrm{yy}} & 0\\ 0 & 0 & 1+\frac{i}{\in_{0}\omega }\sigma_{\mathrm{zz}} \end{array}\right],$$

### 3.6. Numerical Models and Tools—Simulation, Mesh, and Accuracy Considerations

Analytical models are desirable, of course, in order to define mathematically physical behaviors and case studies of integrated Hall Effect into a device or module. However, complementary analysis using numerical models and simulations remain desirable in order to simulate and forecast such behaviors. Several TCAD tools exist and were used along the years, in order to complete the device’s specifications. In the following, we will give several examples of such software platforms and packages.

When considering the design of a Hall structure it is also important to pay attention to the effects of non-ohmic contacts and non-symmetrical patterns on the Hall Voltage. Indeed, it is well established that they contribute to non-linearity with the magnetic field and offset, respectively. Usually these artefacts can be avoided by a proper choice of contact materials and a careful design of symmetric contact patterns, as reported, for instance, by Sander [66].

**Figure 4:**
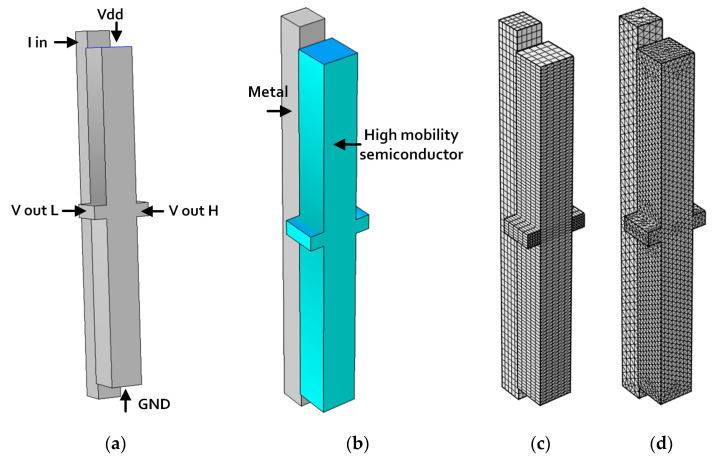
Design flow for a three-dimensional (3D) Hall Bar. (**a**) Preliminary design and definition of ports. (**b**) Layers definition. (**c**) Standard automatic mesh. (**d**) Adapted manual mesh with different density zones, used with tetrahedron default elements or extra-fine elements.

In order to perform a complete and accurate numerical study of such devices, design, and simulations, the platform of Comsol Multi-Physics Software Package [67] is usually used. This platform’s approach is based on the Finite Elements Method (FEM) [68,69], and it shares several modules. Several types of micro- and nanoscale devices have been designed with this platform [70,71]. In the specific case of Hall Amplifier [71], the required simulation models are the AC/DC module, the semiconductors module, and the Heat Transfer module. Even for a simple design such as the Hall Bar (HB) shown in Figure 4, it is necessary to follow a well-built flow of steps such as geometry shapes design, ports and layers definitions, and automatic or manual mesh definition to improve the simulation resolution. Comsol enables several shapes of mesh elements, and an expert designer will know how to optimize the usage of the mesh for better density and accuracy in sensitive parts of the designed structure. Mesh accuracy is very important, since it enables improving the accuracy on the short circuit effect and other secondary effects such as planar Hall Effect, etc. For example, while Figure 4c presents the default automatic mesh structure, made of cubes, Figure 4d presents the same design while manually optimized with triangular smaller elements. Of course, there is a trade-off in such an optimization: Accuracy will always require longer run times since the whole volume is divided in much more elements. This is why it is usually recommended to check first runs with coarse FEM and then to gradually enhance the critical zones’ accuracy. Only then functionality simulations and additional checks can be performed. Sometimes, in order to simulate complex analyses, it is necessary to combine the usage of several additional modules.

Additional FEM-based software packages exist and are used for the purpose of device simulations. For example, the Finite Element Analysis (FEA) of a sensor module was performed using the Flux 2D software [72], and the check of the variation of the sensor module’s leakage flux at two distinct positions A and B was performed with color distribution mapping, describing the magnetic flux density. In another case study, the open source 2D Finite Element Method Magnetics (FEMM) created by Meeker [73] was used for Magnetic Force Modeling [74]. In addition to Comsol, Flux, FEMM, and other kinds of Finite Elements Methods, it is necessary to use, in addition, MATLAB complementary software [75] for the mathematical modeling of the device behavior.

An additional TCAD tool used for the numerical forecast of Hall Effect based devices is the Synopsys Sentaurus TCAD tool [76]. For instance, this three-dimensional platform was used for the comparative study of Hall Effect devices [77]. Sometimes, the TCAD is chosen as a function of the application, since it fits the simulation needs. For example, in the domain of the shape and arrangement of the Hall sensor and magnets for soft fingertip, the model was constructed in Abaqus (Dassault System, Waltham, MA, USA) for simulation [78], using, yet again, the Finite Elements (FE) model.

## 4. Review of Macroscale Hall Effect-Based Devices

Since many Hall Effect-based devices and applications can be found in the literature, it became necessary, for clarity sake, to classify them in three categories: (1) MEMs and macroscale devices (≥1 mm), (2) microscale devices (≥1 µm), (3) nanoscale and quantum-based devices (<100 nm), as reviewed and presented in the following paragraphs, and in the alphabetical summary Table 1, Table 2 and Table 3, respectively. As part of the review, a special effort was invested in the tables’ construction, and in linking existing devices to the relevant Hall Effects reported above, on which they are designed. One can note that the smaller the devices are, the smaller are the lists of devices. The reason for this is that the integration of Hall Effect into the nanotechnology world is quite challenging and limited by several reasons discussed in paragraph 6. In the tables, we tried to expand the list of existing devices, whereas in the review paragraphs, we tried to focus on recent, selected published applications.

### 4.1. Planar Hall Effect (PHE) Sensors

When dealing with macroscale, one can explore a large range of dimensions, from mega-magnets through the Micro-Electro-Mechanical System (MEMS). Our focus in this article is more oriented to MEMS and thinner devices. Since PHE appears a lot in the literature, we will review only a few, representative publications, in order to emphasize the importance of its possible applications. Recently, Grosz et al. [79] reported the fabrication of some elliptical Planar Hall Effect (PHE) sensors. These sensors, made of Permalloy, share a special shape-induced uniaxial anisotropy. Impressive results and resolutions were obtained after optimization of the sensor thickness and of the excitation current amplitude: Magnetic field resolution of 600 pT/√Hz at 1 Hz, and of 1 nT$\sqrt{\mathrm{Hz}}$ at 0.1 Hz. Of course, additional ways of improvement are discussed. In fact, such a PHE device is not completely new and the research on its predecessors was published around three decades ago with micrometric dimensions [80]. Indeed, the team of Van Dau et al. fabricated sensitive magnetic field detection devices based on PHE. When compared to a latter version of the PHE [79], the structure fabricated by Van Dau et al. shares dimensions of 28 µm legs over a distance of 200 µm. The structure is made again of Permalloy, with ultrathin layers of 6 nm on top of additional layers (Table 4) grown by Molecular Beam Epitaxy (MBE). The optimization showed a sensitivity of minimum detectable field below 10 nT.

### 4.2. Soft Skin Sensors (SSS)

Another representative domain in which macroscale sensors are desirable is robotics. For example, several teams recently worked on developing Soft Skin Sensors (SSS) for robotic applications. In 2016, Tomo et al. presented a new version of such Hall Effect-based SSS [81]. A series of devices was fabricated across the years, and Table 4 presents hereby a summary of the most recently developed components.

**Table 1:** Summary table of the main known macro devices with integrated Hall Effect. Classification is per measured quantity/physical parameter.

Type (Measured Quantity)	Definition and Applications	Domain	Year
Angle sensors [82,83]	Contactless sensor conceived for measuring the rotation angle of a shaft. Signal proportional to angular position.	Automotive, Aeronautics	2013, 2001
Position and speed sensors [84,85]	Position and speed control of Brushless Direct Current (BLDC) motors using several Hall sensors inside the stator on the non-driving end of the motor.	Medical, Military, Robotics	2010, 2019
Current sensors [86,87]	Adjustable sensor for currents ranging from µA to kA.	Power	2016, 2018
Curvature Bend sensors [88]	Large curvature bend sensor based on internal Hall Effect sensor in a cable. Feedback needed for analog control.	Robotics, Motion	2016
Flow rate sensors [89,90]	Measure flow rate of fluids or air. Self-service gas stations sharing demand for pumps with remote reading. Monitoring milk yield.	Fluids, Automotive, Farming	2013, 2013
Magnetic Field Components sensors [79,91,92,93]	Hall Effect-based magnetometers and PHE sensors with high resolution.	Biomedical	2018, 2019, 2020, 2013
Position sensors [72,94]	Position in linear motors using magnetic sensors. Office machine sensor for equipment with moving parts such as copiers, fax, printers.	Robotics, Office	2015, 2017
Pressure sensors [95,96]	Pressure measurement, piston position in a high-pressure. Sensor indication that a machine is not at level.	Industrial, Automotive, Control	2011, 2015
Proximity sensors [74]	Linear Proximity Sensors (LSPs) with mid- and low-range measurement capabilities widely used in industrial and non-industrial applications.	Industrial, non-industrial	2016
Target identification, location and movement sensors [97]	Radio Frequency Identification (RFID) technology for identification of road traffic signals, and high accuracy vehicle speed measurement with Hall Effect-based sensor, placed on vehicle wheel.	Automotive	2010
Rounds Per Minute (RPM) sensors [98]	Speed control, motor timing control, zero speed detection, tape rotation, under/over speed detection, disk speed detection, automobile transmission controller, fan movement, shaft rotation counter, bottle counting, radical position indication, drilling machines, linear or rotary positioning, camera shutter position, rotary position sensing, flow-rate meter, tachometer pick-ups.	Automotive	2014
Soft tactile and skin sensors [81,99,100]	Magnetic-based soft skin/tactile sensors. Current detection of several levels.	Robotics	2016, 2019, 2017
Speed sensors [101]	Pipeline Inspection Gauge (PIG) speed based on Hall Effect sensor. Operations number sequencing and/or duration.	Petroleum	2017
Tactile sensors [78,102,103]	Hall Effect-based soft tactile sensors.	Robotics	2019, 2020, 2016
Temperature sensors [104,105]	Temperature measurement. Distributor mounted ignition sensor. Temperature range of −40 to 150 °C. Door electrical interlock for ignition system.	Automotive, Office	2016

## 5. Review of Microscale Hall Effect-Based Devices

As part of our study, we will present a selected review of the main trends in the microscale range. This time, we will compare two types of components and their corresponding materials. Recent applications of such devices are summarized in Table 2.

### 5.1. CMOS Hall Sensors in Silicon

If the macroscale devices are used in a large diversity of domains, such as robotics [103], biomedical [92], medicine [102], astronomy [106], automotive [31], military, farming [89], office [31], etc., it appears that microscale devices are more oriented to be integrated into the microelectronics circuitry and industry. Several studies were performed on the Hall Effect in semiconductors in general, and in Silicon in particular [107]. As an example is the development of the CMOS sensors [77] in which special design rules were defined to create them [108]. Additional studies focused on the design and the integration of Hall sensors into the CMOS 0.35 µm technology, when comparing nine different shapes of devices [109]. The aim was to enable maximal sensitivity as a function of the geometry and the dimensions. More recently, and progressing towards the integration in smaller dimensions, semiconductor-based magnetic sensors such as Hall sensors have been implemented in CMOS 0.18 µm technology, in order to enable a new concept of drain current modeling in rectangular normal MOS transistors [110]. Yet another example is the integration of Hall Effect magnetic sensors in CMOS technology which was already designed and studied twenty years ago [111] to eliminate influences of packaging stress and temperature variations.

CMOS Hall Effect devices are the most studied and produced device (several billions of devices produced every year for the sole automotive sector). In particular, novel devices and signal conditioning techniques to reduce offset and packaging stress in silicon devices have been widely studied and state-of-the-art works were presented [66,112,113]. For example, Sander presented a novel CMOS-integrated device, entitled Vertical Hall Sensor (VHS) [66], sharing an optimized symmetry for in-plane magnetic field components’ measurement. This novel device enabled higher degree of symmetry by using an appropriate connection of four identical three-contact elements. The device led to two important improvements: First, a factor of more than four over the 5CVHS fabricated on the same wafer, and second a power consumption reduction of 47%. Five years later, Frick and Osberger presented a chopper-stabilized MAGFET (CHOPFET) [112], a magneto transistor which is compatible with the spinning current technique for low-frequency noise and offset cancelation. While a prototype was fabricated in the 0.35 μm CMOS process, it could show a minimum value of 0.75 noise correlation between the two consecutive switching phases.

### 5.2. Bipolar PNP Junctions in Graphene

According to the bibliography of a large body of publications, it appears that in addition to the efforts invested in silicon-based devices [77,107,108,109], graphene-based devices are also desirable [114]. Due to its exceptional 2D high electrical mobility and thermal conductivity, graphene could be a game changer in the microelectronics industry, moving from silicon to graphene applications, including Hall Effect integrated devices. Moreover, as reported in the next paragraph, the graphene is a fruitful substrate for new Hall Effects at the nanoscale range, when fabricating nanostructures [115]. Indeed, by looking after electronic transport measurements, fractional quantum Hall conductance plateau were identified in bipolar graphene PNP junctions.

**Table 2:** Alphabetical summary table of the main known micro devices with integrated Hall Effect. Classification is per device acronym.

Type	Definition and Applications	Domain	Year
CMOS sensors [77,108]	CMOS Hall Effect Sensors	Microelectronics	2013, 2017
CHOPFET [112]	Chopper-Stabilized MAGFET	Microelectronics	2018
GHE [114]	Graphene Hall Element	Microelectronics	2013
HEBCS [70]	Hall Effect-Based Current Sensor		2018
µA Hall sensor [116]	Switching function in low-power	Microelectronics	2018
MOS current sensor [110,117]	Power Electronics Converters	Microelectronics	2015, 2017
MOS magnetic sensor [111]	Technique to eliminate influences of packaging stresses and temperature variations	Microelectronics	2001
PHE sensors [80]	Sensitive magnetic field detection	Magnetics	1995
SHEM [118]	Scanning Hall Effect Microscope	Geology	2019
VHS [66]	Vertical Hall Sensor	Microelectronics	2013

## 6. Review of Nanoscale Hall Effect-Based Devices

### 6.1. Hall Effect Sensors (HES)

With the challenging prospective to implement the Hall Effect into the nanotechnology domain, several efforts focused on using the phenomenon as a basis for accurate sensors. Recent applications of such devices are summarized in Table 3. Nano sensors have multiple applications in different sciences such as biomedicine, environment, communications, and production of smart substances. In fact, these studies first focused on research in biology, where the material used for the sensor is graphene [119]. Since graphene is a 2D material composed of carbon atoms which have drawn the attention of researchers not only due to specific properties such as high electron mobility and band gap close to zero, but also due its high biological compatibility, it was natural to move to biology orientation based on graphene substrate. Remaining with a graphene support, the focus recently moved to nanoscale magnetic sensing and imaging [120]. In this reference, graphene Hall sensors have been fabricated using the Chemical Vapor Deposition (CVD) process, sharing wire widths between 50 and 1500 nm, and in order to exploit the high carrier mobility and tuneability of this material.

An additional domain of Hall Effect nanoscale applications is the ultra-microscopy. Around two decades ago, several studies focused on the nano and micro Hall Effect sensors for room-temperature scanning hall probe microscopy. The aim was to develop and fabricate nano and micro Hall Effect sensors using Bi and InSb thin films, and to show how they can be practical alternatives to the GaAs-2DEG probes for Scanning Hall Probe Microscopy (SHPM) [121]. Again, one can observe that applications and materials are running together, when each time the domain fixes the optimal material to be used. Several tries such as MoS_2_ transistors [60], and Hall Effect sensors [122] were also studied. Moving forward with electromagnetic fields in circuitry, ballistic deflection transistors were also studied [123]. In those cases, the ballistic effects in transistors were examined [124,125,126].

### 6.2. Hall Amplifier Nanoscale Device (HAND)

**Figure 5:**
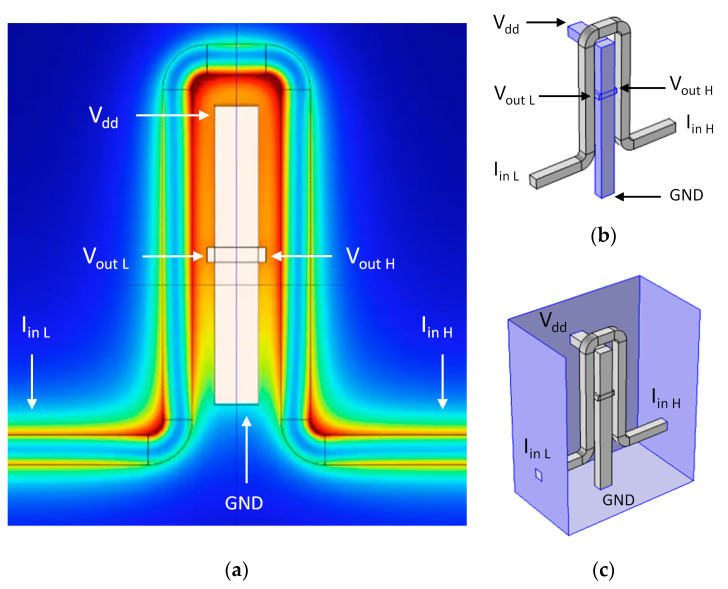
Hall Amplifier Nanoscale Device (HAND) two-dimensional (2D) structure in Comsol. (**a**) With activated field. (**b**) 3D view. (**c**) In simulation box.

**Figure 6:**
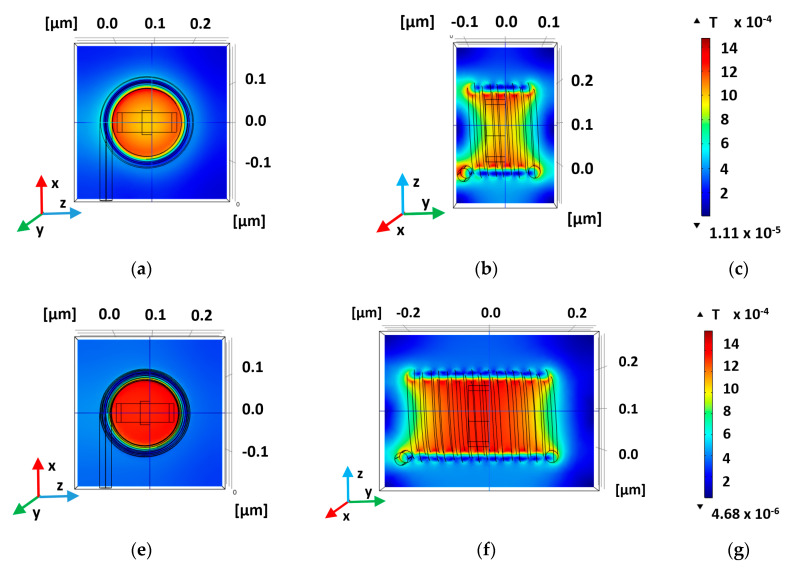
Magnetic flux density Norm (T), produced by a 15 nm copper coil with a number of loops, and an input electric current of 30 μA. (**a**) Face-view, five loops; (**b**) cross-view, five loops; (**c**) scale, five loops; (**d**) face-view, ten loops; (**e**) cross-view, ten loops; (**f**) scale, ten loops.

If Hall Effect sensors are well known devices, additional components such as amplifiers are less famous. Recently, a new device nanoscale component called HAND (Hall Amplifier Nanoscale Device) was designed, simulated, and modeled (Figure 5) [71]. The aim was to enable the integration of the original macro Hall Effect in tiny circuitry compatible with modern silicon processes. Since we can expect ultra-high working frequencies (>10 THz) the HAND device could be a game changer for computing circuits. The design and the numerical checks were all performed using the Comsol Multi-Physics Package Software. Additional efforts were invested in complementary analytical models in order to better understand device functionality. As presented in Figure 6, HAND’S design includes a copper coil, with varying number of loops, surrounding a doped GaAs Hall bar. This material was chosen for its high mobility. The width of the bar is 50 nm. The rationale is to run a high frequency electric current through a surrounding coil, while creating an AC magnetic field inside the Hall Bar itself and, due to the Hall Effect, resulting in an AC voltage between its two knobs. In fact, the Hall Effect amplifier idea is not new, since it already appeared in the fifties [127,128,129]. These references remind us again that although the idea appeared almost seven decades ago, the technology was not advanced enough to fabricate micro- or nanoscale devices to be integrated in the circuitry. Sometimes, there is a prolonged delay between the idea and its realization, due to a lack of suitable technologies and/or relevant materials. The main innovation and application that HAND suggests are the possibility to use nanotechnology knowledge in order to integrate extremely small devices, and to enable terahertz electronic frequencies. Such a device could serve as a revolutionary game changer. In fact, when compared to previous research on ballistic deflation devices, where deflection is conducted with electrical fields, current research looks for an alternative option using magnetic fields.

### 6.3. Hall Quantum-Based Structures

From the moment we reach nanoscale dimensions (1–100 nm), we may expect quantum effects and phenomena occurring in the reduced range of 1–10 nm. In addition to the above nanoscale sensors and amplifiers, several teams worked on the development of quantum-based structures, using also the Hall Effect. One recent example is the development of a Highly Sensitive Nano-Tesla Quantum-Well Hall-Effect integrated circuit (IC) using GaAs-InGaAs-AlGaAs 2D Electron Gas (2DEG) technology. This quantum-based structure shares the name of ultrasensitive Linear Hall Effect Integrated Circuits (LHEICs) [130]. Its performances are quite remarkable since it is capable of detecting AC low magnetic fields as low as 177 nT. When dealing with the quantum dimensions range, one can observe that published studies are more oriented to monolayers “structures” than to applicable devices. There is still a long way to go in order to translate a quantum-based effect into a well working component. Of course, these studies are very important for the understanding of the carriers’ behavior, however they remain in the theoretical domain. An example of such a case is the very recent study of Quantum Valley Hall Effect (QVHE) [131], using SiC monolayer. In such cases, we are dealing with 2D materials of group IV elements, such as graphene, silicene, germanene, and stannene which are monatomic. Another example of structure is the silicon-based Photonic Topological Insulator (PTI) [132], based also on QVHE. Since topological phases of light have been studied in the last decade, the design of a valley Hall all-dielectric PTI emerged as the next phase.

**Table 3:** Alphabetical summary table of the main known nano devices and structures with integrated Hall Effect. Classification is per device acronym and function. One can observe how few are the nanoscale devices when compared to their macro and micro predecessors.

Type	Definition and Applications	Domain	Year	Effect
Amplifier (THz) [71]	Hall Amplifier Nanoscale Device (HAND)	Electronics	2019	OHE
Hall Nano Probes [133]	Magnetometers, active areas < 100 × 100 nm^2^	Imaging	2006	
LHEIC [130]	Linear Hall Effect Integrated Circuit		2015	LHE
LF AHE sensor [134]	Low-Frequency noise AHE magnetic sensor	Electronics	2019	AHE
P3HT-ZnO NW [135]	P3HT-ZnO Nanowires Gas Sensor	Chemistry	2018	
PHRB [136]	Planar Hall Resistance Biosensor	Biology	2020	PHE
PTI [132]	Photonic Topological Insulator structure	Theoretical	2016	QVHE
QVHE structure [131]	Quantum Valley Hall Effect SiC monolayer	Theoretical	2020	QVHE
Sensor [119]	Magnetic field diagnosis and measurement	Biology	2015	
Sensor [120]	High-resolution ambient magnetic imaging	Imaging	2019	

## 7. Forecast, Expected Trends, and Perspectives

This review paper tried to link between phenomena and corresponding devices, as presented above in the summary of Table 1, Table 2 and Table 3. In this last paragraph, we aim to try forecasting the next steps in the evolution of integrated Hall Effects in smart nanoscale devices, and why some of the phenomena still remain difficult challenges to be integrated. After referring hereby to more than 150 recent articles and books, and reviewing a few hundreds more, it appears to us that the great idea to implement the phenomena in applications is not yet straight forward, and at least six main challenges are still remaining. Tens of groups are still working across the world in order to pass these challenges. The main reason is that the need and the global market for sensors based on magnetic fields are very high, as described later.

### 7.1. Review of the Nanoscale Challenges

#### 7.1.1. Choice of the Material: Dimensions, Application, and Integration

Choosing the material remains the first question to be solved when designing a Hall Effect-based device. As reviewed above, several elements and material candidates have been chosen across the years: Silicon, graphene, GaAs and doped GaAs, InGaAs, AlGaAs, permalloy, copper, etc. The decision on the element or the material will mainly depend on three main parameters: (1) The required dimensions of the planned device, (2) the application to be realized, and (3) how smooth can these two—material and device—be integrated into the existing industry. From macro- to nano-, through microscales, the aspiration is to enable a smooth integration into existing technologies. For example, smart development and integration of new devices into the microelectronics world and CMOS technology will require silicon-based devices. As per Dankert et al. [137], there are three material candidates in the microelectronics world in order to realize Hall sensors: Silicon, III-V compound semiconductors, and graphene. Due to the low cost in the fabrication and the possible smooth integration into CMOS technology, the Hall silicon sensors (using an active region) are omnipresent in the market. As a second choice, enabling much better performances [138], the III-V compound semiconductors are still very difficult to integrate in an old well-established silicon industry. The same dilemma exists for the electro-optics and photonics devices. At the end, graphene became an interesting material to serve as active regions for magnetic sensors, due to its specifications: 2D nature, low carrier concentration, and high carrier mobility. Since the Hall Effect applications were studied in several types of elements and materials across the literature, Table 4 presents a summary of the materials used in this domain.

**Table 4:** Summary table of materials and elements used for Hall Effect studies across the literature.

Symbol/Formula	Name	Main References
Al	Aluminium	[31]
AlGaAs	Aluminium Gallium Arsenide	[130]
Fe	Iron	[80]
Fe-Pt	Iron-Platinium ferromagnetic alloys	[134]
Ga	Gallium	[71,116]
GaAs	Gallium Arsenide	[71,116]
GaAs-InGaAs-AlGaAs	Gallium Arsenide—Indium Gallium Arsenide—Aluminum Gallium Arsenide	[130]
C_140_H_42_O_20_	Graphene	[114,119,120]
h-BN/graphene/h-BN	Graphene hetero-structures	[137]
InGaAs	Indium Gallium Arsenide	[130]
InSb	Indium Antimonide	[121]
Mg	Magnesium	[80]
MgO	Magnesium Oxide	[80]
Nd-Fe-B	Neodymium magnet coated with Nickel	[81]
Ni	Nickel	[80]
Ni-Fe-Mo	Permalloy (usually 80% nickel, 20% iron)	[79,80]
Pd	Palladium	[80]
P3HT-ZnO	Zinc Oxide (ZnO) nanowire array fabricated by Atomic Layer Deposition and organic material p-type semiconductor poly(3-hexylthiophene) (P3HT)	[135]
Si	Silicon	[77,80,108,109]
Ta/NiFe/Cu/IrMn/Ta/Si	Ta (5 nm)/NiFe (10 nm)/Cu (x = 0~1.2 nm)/IrMn (10 nm)/Ta (5 nm)/Si substrate	[136]

#### 7.1.2. Classic limitations: Mega-Magnet, High Temperature, and Self-Heating

In some case studies, for example in nanotechnology circuitry, one can face a challenging trade-off choice between the need for physical high magnetic field, i.e., mega-magnet, and, on the other side, the selected application, in which the device will be implemented, and in which one cannot allow such a mega-magnet. With the same rationale stating one cannot allow high amplitude magnetic field (>0.5 T) inside the circuit, it is also not recommended to use high temperature, which may affect the circuitry’s functionality and performance. In the world of microelectronics and nanotechnology one cannot allow a high temperature inside the circuits, as it can cause device degradation and affect the circuit performances, or cause a self-heating non-desirable phenomenon. When dealing with circuitry and devices, it seems that good functionality is not a good enough reason to claim that the component is successful. Quality and Reliability (Q&R) concerns and considerations should be analyzed and predicted as well. Moreover, when reaching the micro- and nanoscales, additional phenomena may occur. For example, in case of Hall Bar (HB) made of metal, the high temperature phenomenon mentioned above can create degradation mechanisms such as Electro-Migration (EM) [139,140], and Self-Heating (SH) [141], such as in metal interconnects in the VLSI technology.

In spite of the above listed classic limitations, nanotechnology and mega-magnets will not necessarily always remain incompatible or will not be able to share the same application. As a first example, we can consider the bio-medical imaging applications for which heavy medical equipment can share mega-magnets and magnetic resonance, working in harmony with a series of nano-sensors located in separated areas. Such applications usually focus on improving the quality of the imaging process and the results analysis of tested samples or people. In such mega-systems, both required mega-magnets and series of nano-sensors will work in harmony, in spite of being separated from each other. An additional and similar domain, combining both sides of the trade-off, is the equipment needed for the study of particles, and pure physics. It is a fair expectation that in the not so far future similar analyzing equipment will be smaller in size and become portable for mobile applications.

Taking a leap ahead, another example of desirable combined classic limitations (mega-magnet, high temperatures) and nanotechnology, is suitable. Looking again at the Hall Amplifier Nanoscale Device (HAND), cited above [71], one could observe that it cannot tolerate high temperatures. For this specific device, in order to solve the problem of high temperature generated by extremely high current density in the copper nanowire, which surrounds the hall bar, one can suggest several possible optimization paths: Dimensions, materials, coils loop density, and geometry. There also may be trade-off concerns and considerations. For example, increasing the electromagnet cross-section area, for larger sustained current and stronger magnetic field. On the other hand, keeping the device small enough is necessary in order to keep the nanoscale advantage. Therefore, if the optimization of the device’s size may be the first direction to check, usage of alternative materials, enabling higher current densities, could be part of the solution in this dilemma. In the field of superconductors, they may help in producing the magnetic field needed, this time with lower generated temperatures. Geometry changes in the parts of the device can be also a solution. For example, the magnetic field gets stronger as the number of loops increases (Figure 6a–f), and more uniform around the Hall Bar than for single loop coil case study. The maximal field is in range of 1 mT for five loops and up to 2 mT for ten loops [71]. In order to produce a stronger magnetic field with less current density, additional solutions may be checked.

#### 7.1.3. Quantum Limitations and Ballistic Models

When dealing with nanoscale, one cannot ignore additional phenomena such as ballistic effects, carriers additional transport effects, and quantum effects. Sometimes, preliminary analytical developed models may require additional analyses, enhancement, and adaptation, as for the HAND [71], by including more transport considerations. Several complementary models can support these enhancements. For example, the Kubo-Greenwood-Chester-Thellung formulation [65,142] is suitable for quantum transport investigations in disordered bulk materials. However, it may be incomplete to simulate nanoscale devices approaching the ballistic regime. An additional approach such as Landauer-Büttiker Formalism [142,143,144,145] is requested for contact effects and non-equilibrium transport properties. The quantum Hall Effect is not considerable if the magnetic field will be less then B < 0.5T [145]. Superconductors could possibly allow stronger magnetic field with less emitted heat, and enable quantum Hall Effect relevance to Hall Amplifier operation.

#### 7.1.4. Fabrication and Smooth Integration

Fabrication of nanoscale structures and devices is one of the main challenges for several reasons. First, there are huge differences between the laboratory process for research purpose and industrial fabrication for massive production. The equipment, the standards, the mode of work, the expectations, qualification, and testing parts are not comparable. While scientific laboratories and nanotechnology centers are usually interested in a proof of concept for a new structure, mainly focusing on the functionality aspect, high-tech industries are looking forward for quality and reliability (Q&R) criteria of their fabricated devices. Second, there are yet not enough production plants with nanoscale range accuracy and capability. If the process flow of macroscale Hall Effect devices, such as in automotive and robotics applications, is well established, such industrial process flows for the nanoscale range do not exist as of yet or are not mature enough. Moving to the deposition of thin layers for smaller devices, some standard equipment has been successfully used in the past. About two decades ago, Boero et al. already reported the usage of several techniques as a function of the elements used [146] for micro devices. For example, Molecular Beam Epitaxy (MBE) or metallo-organic chemical vapor deposition (MOCVD), by optical, electron beam, and ion beam were used for the realization of GaAs and InSb heterostructures. Chemical Vapor Deposition is frequently used today [120,137] for graphene. As for nanoscale devices, Focused Ion Beam (FIB) was shown useful for the fabrication of patterning gold and Si-doped GaAs nano-probes, as reported above [133]. The focused electron-beam-induced was used for sub-micron Hall devices [147] as reported by Boero et al.

### 7.2. Expected Trends

As per *MarketAndMarket* recent study [148], the Hall Effect current sensor market is a huge one. For example, between the years 2016 to 2023, the expected growth is from USD 831.0 to 1473.7 million, with a compound annual growth rate (CAGR) of 8.4%. Several parameters, in addition to the growing demand for such devices, can serve as a catalyst to help this market grow in the following years.

In addition, the nanotechnology-based medical devices and applications market is also expanding [149], valued at approximately $5 billion in 2014, and was expected to reach around $8.5 billion by 2019 with a compound annual growth rate (CAGR) of around 11–12% during the forecast period of 2014–2019. As reported there, “The rising aging population, increasing R&D expenditure, and international research collaborations, mainly drives this market. However, high costs of nanotechnology-based medical devices and time-consuming product approval processes are inhibiting the growth of this market to a certain extent.” It is reasonable to assume that combining efforts to develop nanoscale devices, based on the Hall Effects family, will provide a big leap ahead in the coming years. For this reason, it may be recommended that efforts be invested in nanoscale device development, not only structures, but also and mainly component development.

## 8. Conclusions

An extended up-to-date review of the Hall Effect-based devices, circuitry, and PDKs was presented with sub-classifications to macro-, micro-, nano-, and quantum-based scales. Since nanotechnology is today one of the main important domains for new generations of computing and instrumentation, the current review may serve as an adequate groundwork for scientific community members. In spite of the trade-off considerations and of the remaining challenging barriers, both classic and ballistic, Hall Effects and nanoscale devices are merging to one important path, delivering advanced sensing devices. In the coming decade, it is more than plausible to expect smart applications in which sensors, amplifiers, switches, and other devices will appear in integrated platforms.

## Figures and Tables

**Figure A1:**
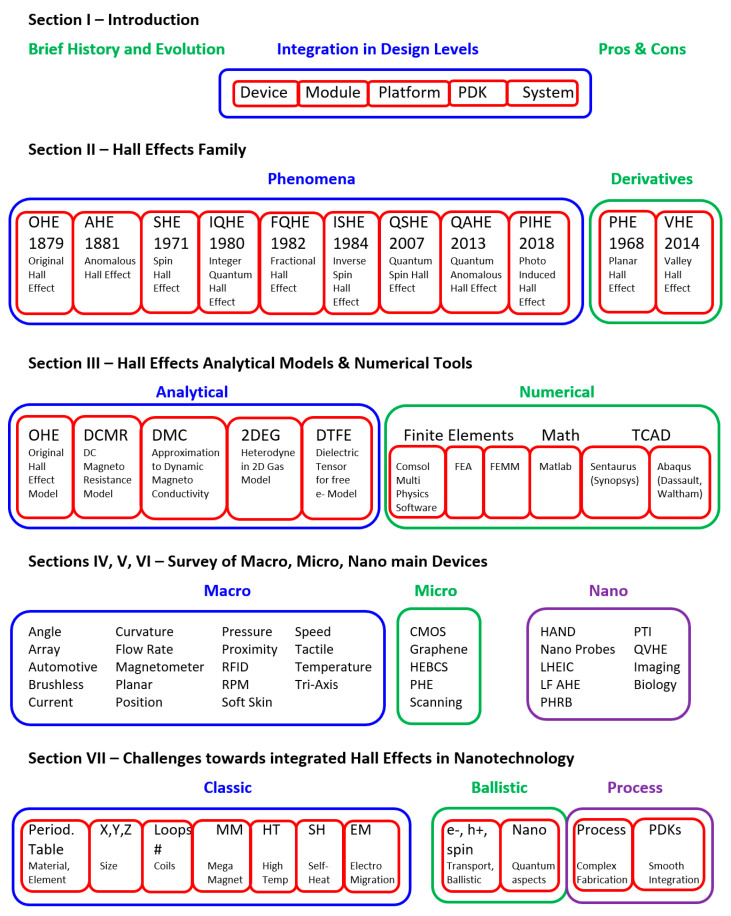
Condensed overview of this survey.

## Appendix A. Electro-Magnetic Parameters and Units

Hall Effect appears in electro-magnetic fields. The Table 1 presents a summary of the main physical parameters and units used in this domain.

**Table A1:** Summary table of the electrical, magnetic, and dimensional parameters and units.

Quantity	Symbol	Unit Name (SI)	Unit
**Electrical parameters and quantities:**			
Electric Field	$\vec{E}$	Volt/meter	V/m
Current	I	Ampere	A
Charge	q	Coulomb	C
Voltage	V	Volt	V
Power	P	Watt	W
Resistance	R	Ohm	Ω
Impedance	Z	Ohm	Ω
Capacitance	C	Farad	F
Inductance	L	Henry	H
Frequency	f	Hertz	Hz
Period	T	Seconds	s
**Magnetic parameters and quantities:**			
Magnetic Field	$\vec{B}$	Tesla	T
Magnetic Flux	Φ	Weber	Wb
Magnetic Field Strength	H	Ampere/meter	A/m
Permeance	P	Henry	H
**Dimension parameters and quantities:**			
Length	L	Meter	m
Mass	m	Kilogram	Kg
Time	t	Second	s

## Appendix B. Hall Effect Acronyms

Tens of Hall Effect related acronyms have been cited in this article. As complementary information to this study, Table A2 is presented below in order to enable quick search.

**Table A2:** Summary table of the Hall Effects related acronyms.

Acronym	Definition	Domain
2DEG	2D Electron Gas	Physical Scale
DOF	Degree of Freedom	Physical Parameter
DSHE	Direct Spin Hall Effect	Physical Effect
EM	Electro-Migration	Physical Effect
FE	Finite Elements	Software
FEM	Finite Element Method	Software
FEMM	Finite Element Method Magnetics	Software
FQHE	Fractional Quantum Hall Effect	Physical Effect
GHE	Graphene Hall Element	Device
HAND	Hall Amplifier Nanoscale Device	Device
HB	Hall Bar	Device
HE	Hall Effect	Physical Effect
HES	Hall Effect Sensors	Device
HEBCS	Hall Effect-Based Current Sensor	Device
IQHE	Integer Quantum Hall Effect	Physical Effect
HV	Hall Voltage (V_H_)	Physical Parameter
ISHE	Inverse Spin Hall Effect	Physical Effect
LHEIC	Linear Hall Effect Integrated Circuit	Circuit
LPS	Linear Proximity Sensor	Device
MBE	Molecular Beam Epitaxy	Technology
MEF	Magnetic and Electric Fields	Physical Effect
MEMS	Micro-Electro-Mechanical System	Circuit
OHE	Original Hall Effect, Ordinary Hall Effect	Physical Effect
PDK	Process Development Kit	Technology
PHE	Planar Hall Effect (Sensor)	Device
PIHE	Photo-Induced Hall Effect	Physical Effect
PTI	Photonic Topological Insulator	Structure
Q&R	Quality and Reliability	Physical Area
QAHE	Quantum Anomalous Hall Effect	Physical Effect
QHE	Quantum Hall Effect	Physical Effect
QHS	Quantum Hall State	Physical Effect
QSHE	Quantum Spin Hall Effect	Physical Effect
QVHE	Quantum Valley Hall Effect	Physical Effect
QW	Quantum Well	Device
SH	Self-Heating	Physical Effect
SHE	Spin Hall Effect	Physical Effect
SHPM	Scanning Hall Probe Microscope	Physical Area
SSS	Soft Skin Sensor	Device

## Appendix C. Condensed Overview of this Survey

In order to enable a quick one-page summary of this survey, Figure A1 presents the main key learning points per section.
